# Prognostic significance of somatic mutations in myeloid cells of men with chronic heart failure – interaction between loss of Y chromosome and clonal haematopoiesis

**DOI:** 10.1002/ejhf.3778

**Published:** 2025-07-24

**Authors:** Sebastian Cremer, Moritz von Scheidt, Klara Kirschbaum, Lukas Tombor, Silvia Mas‐Peiro, Wesley Abplanalp, Tina Rasper, Akshay Ware, Andrin Schuff, Alexander Berkowitsch, Johannes Krefting, David Leistner, Heribert Schunkert, Thimoteus Speer, Stefanie Dimmeler, Andreas Michael Zeiher

**Affiliations:** ^1^ Department of Medicine, Cardiology Goethe University Hospital Frankfurt Germany; ^2^ Institute for Cardiovascular Regeneration, Goethe University Frankfurt Germany; ^3^ German Center for Cardiovascular Research, partner site Frankfurt Rhine‐Main Berlin Germany; ^4^ Cardiopulmonary Institute, Goethe University Frankfurt Frankfurt Germany; ^5^ German Heart Center Munich Germany; ^6^ Department of Medicine Nephrology, Goethe University Hospital Frankfurt Germany; ^7^ Else Kroener‐Fresenius Center for Nephrological Research, Goethe University Frankfurt Germany

**Keywords:** Inflammation, Bone marrow, CHIP, Heart failure

## Abstract

**Aims:**

Age‐associated clonal haematopoiesis of indeterminate potential (CHIP) has been linked to increased incidence and worse prognosis of chronic heart failure (CHF). CHIP arises from somatic mutations in haematopoietic stem and progenitor cells. Mosaic loss of Y chromosome (LOY), the most common somatic mutation in male blood cells, increases with age, drives clonal expansion of myeloid cells, and has been experimentally associated with cardiac fibrosis and heart failure in mice. However, its prognostic value and interplay with CHIP in CHF patients remain unclear.

**Methods and results:**

We analysed 781 male CHF patients across the full spectrum of left ventricular ejection fraction to assess the prevalence and prognostic relevance of LOY and the two most common CHIP‐driver mutations, DNMT3A and TET2. Both LOY and CHIP mutations increased with age and co‐occurred in 27.1% of men >70 years. LOY independently predicted all‐cause mortality in patients with heart failure with reduced ejection fraction (HFrEF). The co‐occurrence of LOY and DNMT3A/TET2 mutations further increased mortality among CHIP carriers. This detrimental prognostic effect of LOY was confirmed in a validation cohort of HFrEF patients. Single‐cell RNA sequencing of peripheral blood mononuclear cells from HFrEF patients with ischaemic heart failure revealed elevated pro‐fibrotic signalling in LOY monocytes, characterized by increased inflammatory and remodelling markers (S100A8, TLR2, CLEC4D) and decreased expression of transforming growth factor‐β inhibitors (SMAD7, TGIF2). In patients with both LOY and DNMT3A mutations, monocytes showed enhanced pro‐inflammatory gene expression, including alarmins (S100A8, HMGB2) and interferon‐related genes (IFNGR1, TRIM56, CD84).

**Conclusions:**

Somatic mutations in blood cells—particularly LOY—are associated with increased mortality in male CHF patients, with LOY emerging as an independent prognostic marker.

## Introduction

Somatically acquired DNA mutations in haematopoietic stem cells have recently been identified as independent risk factors for the development and progression of cardiovascular disease.^1^ Most notably, clonal haematopoiesis of indeterminate potential (CHIP), defined as the presence of mutations in genes commonly associated with myeloid neoplasms in peripheral blood cells of subjects without any evidence for haematological malignancies, was previously shown to associate with the incidence and progression of chronic heart failure.^2^, ^3^, ^4^, ^5^, ^6^, ^7^ Specifically TET2 mutations also associate with incident heart failure with preserved ejection fraction (HFpEF), as just recently reported.^8^, ^9^ CHIP increases with age, confers a proliferative advantage leading to clonal expansion of mutant blood cells and is detected in >20% of patients with cardiovascular disease above the age of 70 years.^2^, ^10^, ^11^ On the other hand, mosaic loss of Y chromosome (LOY), defined as loss of the entire Y chromosome in a mosaic fashion, is the most common acquired somatic mutation in human blood cells and detected in >40% of men above the age of 70 years in the UK Biobank.^12^, ^13^ Similar to CHIP, LOY increases with age and correlates with clonal expansion of myeloid cells.^12^, ^14^ Most importantly, Sano *et al*.^15^ recently experimentally demonstrated that haematopoietic LOY in mice led to diffuse cardiac fibrosis during ageing culminating in the development of heart failure.

We could recently demonstrate that LOY in blood cells is associated with profoundly impaired long‐term survival even after successful removal of the stenotic aortic valve in men undergoing transcatheter aortic valve replacement (TAVR).^16^ However, the prognostic significance of LOY in established chronic heart failure has not been assessed so far. Moreover, given the similarities in age‐related prevalence and cardiovascular disease risk, it is tempting to speculate that LOY and CHIP may represent two sides of the same coin.^17^

Therefore, in the present study, we investigated both, the prognostic significance of LOY as well as its potential interaction with CHIP in men with chronic heart failure.

## Methods

### Study cohort

In this institutional review board approved study, clinical data and peripheral blood cells were collected from a total of 781 male patients with established heart failure between November 2001 and March 2022. Patients were eligible for inclusion into the present analysis, if they suffered from stable chronic heart failure symptoms (New York Heart Association [NYHA] class ≥II), clinical evidence of heart failure and elevated N‐terminal pro‐B‐type natriuretic peptide (NT‐proBNP) serum levels. Heart failure with reduced ejection fraction (HFrEF) was defined as left ventricular ejection fraction (LVEF) <50%. Exclusion criteria were the presence of acutely decompensated heart failure with NYHA class IV, an ischaemic cardiac event or interventional procedure within 30 days prior to inclusion, documented haematological disease or cancer within the preceding 5 years, or unwillingness to participate. The CHIP results of some of the patients with HFrEF have been previously reported.^2^ Coronary artery disease was defined as prior myocardial infarction or revascularization. In addition, some of the patients reported in our most recent study describing a prognostic significance of LOY after TAVR^16^ were also included, if they suffered from continuous heart failure NYHA class ≥II symptoms and elevated NT‐proBNP serum levels regardless of reduced ejection fraction at least 30 days after successful valve replacement. The primary endpoint of the study was all‐cause mortality during a prospective 3‐year follow‐up.

Validation of the results was performed in an independent external replication cohort at the German Heart Centre Munich. A total of 2003 patients with confirmed coronary artery disease undergoing coronary angiography between January 2014 and December 2022 were screened for LOY status, and all 706 patients with ejection fraction <50% were included in the analyses.

The study complied with the requirements of the Declaration of Helsinki. All participants provided written informed consent for the study procedures, including genetic testing using blood samples.

### Measurement of loss of Y chromosome

Estimation of LOY was performed using a previously validated digital polymerase chain reaction (PCR) technique.^14^ In brief, the TaqMan‐based method quantifies the relative number of X and Y chromosomes in a DNA sample by targeting a 6 bp sequence difference present between the AMELX and AMELY genes using the same primer pair and, thus, is relatively unbiased with regard to primer properties. DNA was extracted from whole blood at the timepoint of study inclusion.

As previously described,^16^ 150 nanograms of DNA was mixed with Probe PCR Master Mix (QIAcuity Probe PCR Kit, #250101, Qiagen, Hilden, Germany) containing FastDigest HindIII enzyme (#FD0504, ThermoFisher Scientific, Waltham, MA, USA) and TaqMan Primers (#C_990000001_10, ThermoFisher Scientific, Waltham, MA, USA). After digestion for 10 min at room temperature, a 26 k 24‐well Nanoplate (QIAcuity Nanoplate 26 k 24‐well, #250001, Qiagen, Hilden, Germany) was filled with the reaction mix and loaded into a QIAcuity One instrument (Qiagen, Hilden, Germany). PCR cycling was performed following manufacturer's instructions: PCR initial heat activation 95°C for 2 min, followed by two‐step cycling (40 cycles) of 95°C for 15 s and 60°C for 30 s. A 6 nt deletion occurs in the X‐specific *amelogenin* gene (B37/hg19 genome locations: chrX:11315039 and chrY:6 737 949–6 737 954). The VIC dye probe detects X‐chromosome sequences, and the FAM dye probe includes the 6 nt and detects Y‐chromosome sequences (Sequence: GTGTTGATTCTTTATCCCAGATG[‐/AAGTGG]TTTCTCAAGTGG TCCTGATTTT [VIC/FAM]).

The endpoint fluorescence intensity of the partitions was separately measured for FAM (targeting AMELY) and VIC (targeting AMELX) to determine the presence or absence of the respective targets. Using the QIAcuity One Software Suite (Qiagen, Hilden, Germany), the absolute concentration of the targets was calculated based on the number of positive and negative partitions. The ratio of AMELY/AMELX was calculated using absolute concentrations. The extent of LOY as percentage was defined as the ratio of AMELY copy numbers in relation to AMELX copy numbers and calculated by the formula: 1−(Copy numbers AMELY/Copy numbers AMELX) × 100.

### Sequencing for DNMT3A and TET2‐mediated clonal haematopoiesis

The presence of somatic *DNMT3A* or *TET2* CHIP‐driver mutations with a variant allele frequency (VAF) ≥2% in patients with aortic stenosis as well as in the replication cohort was commercially assessed by next generation sequencing, as extensively described previously.^18^, ^19^ In brief, the patients' libraries were generated with the Nextera Flex for enrichment kit (Illumina, San Diego, CA, USA) and sequences for DNMT3A and TET2 enriched with the IDT xGen hybridization capture of DNA libraries protocol and customized probes (IDT, Coralville, IA, USA). The libraries were sequenced on an Illumina NovaSeq 6000 with a mean coverage of 2147× and a minimum coverage of 400×, reaching a sensitivity of 2%. Reads were mapped to the reference genome (UCSC hg19) using Isaac aligner (v2.10.12) and a small somatic variant calling was performed with Pisces (v5.1.3.60). Protein truncating variants were classified as mutation. Non‐synonymous changes were included, if they were well annotated (several definite submissions to COSMIC, IRAC, or ClinVAR). Other non‐protein truncating variants were defined as variants of uncertain significance.

In patients with ischaemic HFrEF, a custom panel based on the Illumina TruSeq Custom Amplicon Low Input assay was designed to assess the presence of CHIP, including 594 amplicons in 56 genes commonly mutated in CHIP and myeloid malignancies. The pooled libraries were sequenced on a NextSeq 500 sequencer (Illumina, San Diego, CA, USA) using the NextSeq 500/550 Mid Output v2 kit (300 cycles) according to the manufacturer's instructions. The median coverage across all samples was 4282× before unique molecular identifier (UMI) family clustering and 630× with inclusion of UMIs resulting in a sensitivity of 0.5%.^2^

Clonal haematopoiesis of indeterminate potential variants for DNMT3A and TET2 can be found in online supplementary *Tables Appendix* *S1* and *S2*, respectively.

### Single‐cell RNA sequencing analyses

Single‐cell RNA sequencing (scRNA‐seq) was performed as previously described.^20^ In brief, patient‐derived blood was centrifuged on density gradient centrifugation (Pancoll human, Density: 1.077 g/ml, #P04‐60500, PAN‐Biotech GmbH, Aidenbach, Germany), and mononuclear cells were used for droplet scRNA‐seq using the Chromium Controller with Chromium Next GEM Single Cell 3′ GEM, Library and Gel Bead Kit version 3.1 reagent (10× Genomics, Pleasanton, CA, USA) according to the manufacturer's protocol. Libraries were sequenced using paired‐end sequencing by GenomeScan (Leiden, The Netherlands), and expression data were processed by the Cell Ranger Single Cell Software Suite version 3 (10× Genomics, Pleasanton, CA, USA) and aligned to the human reference genome GRCh38. Data integration was performed by Seurat version (v4.0.3) (Satija Lab, New York Genome Center, New York City, NY, USA), and FindMarkers function in the Seurat package was used for statistical analysis of differential gene expression. LOY cells were defined in scRNA‐seq data as combinatorial lack of expression of all Y chromosome‐derived genes. Relative changes in transcription of Y harbouring and LOY cells were then assessed and data were pooled. For subsequent analyses, relative changes in transcription of Y harbouring and LOY cells within each patient were assessed, allowing for paired analyses in patients with DNMT3A driver mutations and without CHIP mutations.

### Statistical analyses

Categorical variables are presented as numbers and frequencies (%), and continuous variables as median (interquartile range [IQR]) unless otherwise noted. We used Cochran–Armitage tests for trend to compute significance values for linear trends of categorical variables over increasing LOY quartiles. Continuous variables were compared between groups by the Wilcoxon rank sum test. The best cut‐off value for the extent of LOY in predicting mortality was calculated using the Youden index^21^ based on the area under the curve from a smooth receiver operating characteristic curve estimation for right censored data. Confidence intervals (CI) and standard errors were estimated with a bootstrapping approach.^22^ For mortality analysis, LOY data were dichotomized according to the cut‐off value to define the groups of patients with high versus low LOY. Three‐year survival was displayed as Kaplan–Meier plots with log‐rank statistic. Univariate and multivariate Cox regression analyses were used to model predictor influences on mortality. Adjustment for the origin of sub‐cohorts (HFrEF or post‐TAVR) was performed by stratified Cox models with the respective cohorts added as stratified variables. All covariables reaching a *p*‐value <0.05 in univariate analysis were included in multivariate analysis to adjust for potential confounding effects. Data analysis was performed with R, version 4.2.1.

Single‐cell RNA sequencing analysis was performed by using R (version 4.0.3) and the analysis tool Seurat (v4.0.3). In brief, differential expression of genes was used utilizing the ‘FindMarkers’ function in the Seurat package for focused analyses. Significant comparisons were performed using the Wilcoxon rank sum test followed by Bonferroni correction. Significance was reported if adjusted *p* < 0.05. Differential transcriptional profiles by LOY status were generated in Seurat with associated gene ontology terms derived from the functional annotation tool Metascape (v3.5) for further analyses.

## Results

### Patient characteristics

The clinical characteristics and laboratory values of the patient cohorts are summarized in *Tables* *1*, *2*, *3*. Patients had a mean age of 74 (62–81) years. The extent of LOY in blood cells ranged from −13.3% to 83.4%. Calculation of the Youden index revealed an optimal cut‐off value for LOY of 17% to predict mortality during 3 years of follow‐up. A total of 141 patients (18.1%) had LOY ≥17% (*Table* *1*). A total of 93 patients (12%) had DNMT3A CHIP‐driver mutations with a VAF ≥2%, 80 patients (10%) had TET2 mutations with a VAF ≥2%, and 10 patients had both DNMT3A and TET2 CHIP‐driver mutations. Altogether, 163 (21.3%) patients harboured DNMT3A and/or TET2 CHIP‐driver mutations (*Table* *2*). The median VAF of patients with DNMT3A mutations was 5.2% (IQR 3.37–12.3%), and the median VAF of patients with TET2 mutations was 5.45% (IQR 3.27–11%).

**Table 1:** Baseline characteristics of all patients and patients with loss of Y chromosome <17% compared to ≥17%

Characteristic	Total (*n* = 781)	LOY <17% (*n* = 640)	LOY ≥17% (*n* = 141)	*p*‐value
Cohort				
Ischaemic heart failure	372 (48)	374 (58)	35 (25)	<0.001
TAVR	409 (52)	266 (42)	106 (75)	
DNMT3A VAF ≥2	93 (12)	77 (12)	16 (11)	0.8
TET2 VAF ≥2	80 (10)	58 (9.1)	22 (16)	0.02
DNMT3A and/or TET2 VAF ≥2	163 (21.3)	127 (20)	36 (26)	0.13
COPD (*n* = 777)	86 (11)	69 (11)	17 (12)	0.7
Diabetes mellitus (*n* = 778)	245 (31)	206 (32)	39 (28)	0.3
Coronary artery disease (*n* = 777)	661 (85)	551 (87)	110 (78)	0.009
Hypertension (*n* = 778)	596 (77)	487 (76)	109 (77)	0.8
Atrial fibrillation (*n* = 695)	439 (57)	360 (57)	79 (56)	0.8
History of smoking (*n* = 544)	347 (64)	295 (63)	52 (67)	0.6
Age, years	74 (62–81)	72 (59–80)	81 (75–83)	<0.001
BMI, kg/m^2^ (*n* = 750)	26.8 (24.6–29.7)	27.0 (24.6–29.7)	26.1 (24.4–29.0)	0.058
LVEF, %	40 (30–55)	40 (28–52)	45 (35–60)	<0.001
<50%	512 (66)	439 (69)	73 (52)	
≥50%	269 (34)	201 (31)	68 (48)	
Creatinine (*n* = 699)	1.13 (0.96–1.41)	1.12 (0.95–1.36)	1.20 (0.96–1.57)	0.12
CRP (*n* = 723)	0.30 (0.14–0.88)	0.31 (0.15–0.86)	0.28 (0.10–0.94)	0.3
Haemoglobin (g/dl) (*n* = 777)	13.50 (12.00–14.70)	13.80 (12.20–14.90)	12.70 (11.10–14.00)	<0.001
Thrombocytes (/nl) (*n* = 726)	204 (167–249)	204 (167–250)	206 (167–243)	0.7
Leucocytes (/nl) (*n* = 776)	7.27 (6.04–8.78)	7.31 (6.09–8.81)	6.96 (5.97–8.31)	0.056
hs‐Troponin (pg/ml) (*n* = 441)	25 (16–47)	25 (16–47)	27 (17–44)	0.7
NT‐proBNP (pg/ml) (*n* = 559)	1367 (542–3289)	1254 (491–3107)	1654 (787–3640)	0.024
ACEi/ARB (*n* = 780)	609 (78)	493 (77)	116 (82)	0.2
ARNI (*n* = 781)	69 (9)	58 (9)	11 (8)	0.633
Aldosterone antagonist (*n* = 752)	275 (37)	241 (39)	34 (25)	0.001
Beta‐blocker (*n* = 750)	575 (77)	477 (78)	98 (71)	0.082
SGLT2i (*n* = 781)	66 (8)	54 (8)	12 (9)	0.997
Statin (*n* = 753)	617 (82)	502 (82)	115 (83)	0.6

Values are presented as *n* (%), or median (interquartile range).

ACEi, angiotensin‐converting enzyme inhibitor; ARNI, angiotensin receptor–neprilysin inhibitor; BMI, body mass index; COPD, chronic obstructive pulmonary disease; CRP, C‐reactive protein; hs‐high‐sensitivity; LOY, loss of Y chromosome; LVEF, left ventricular ejection fraction; NT‐proBNP, N‐terminal pro‐B‐type natriuretic peptide; SGLT2i, sodium–glucose co‐transporter 2 inhibitor; TAVR, transcatheter aortic valve replacement; VAF, variant allele frequency.

**Table 2:** Baseline characteristics of all patients and patients with DNMT3A/TET2 mutations compared to patients without DNMT3A/TET2 mutations

Characteristic	Total (*n* = 781)	No DNMT3A/TET2 (*n* = 618)	DNMT3A/TET2 (*n* = 163)	*p*‐value
Cohort				
Ischaemic heart failure	372 (48)	360 (58)	49 (30)	<0.001
TAVR	409 (52)	258 (42)	114 (70)	
DNMT3A VAF ≥2	93 (12)	0 (0)	93 (57)	<0.001
TET2 VAF ≥2	80 (10)	0 (0)	80 (49)	<0.001
DNMT3A and or TET2 VAF ≥2	163 (21.3)	0 (0)	163 (100)	<0.001
LOY				
<17%	640 (82)	513 (83)	127 (78)	0.13
≥17%	141 (18)	105 (17)	36 (22)	
COPD (*n* = 777)	86 (11)	67 (11)	19 (12)	0.7
Diabetes mellitus (*n* = 778)	245 (31)	187 (30)	58 (36)	0.2
Coronary artery disease (*n* = 777)	661 (85)	529 (86)	132 (82)	0.2
Hypertension (*n* = 778)	596 (77)	470 (76)	126 (78)	0.6
Atrial fibrillation (*n* = 695)	439 (57)	355 (58)	84 (52)	0.2
History of smoking (*n* = 544)	347 (64)	289 (65)	58 (59)	0.3
Age, years	74 (62–81)	72 (60–80)	80 (73–84)	<0.001
BMI, kg/m^2^ (*n* = 750)	26.8 (24.6–29.7)	26.9 (24.6–29.7)	26.7 (24.4–29.2)	0.6
LVEF, %	40 (30–55)	40 (28–50)	45 (32–60)	<0.001
<50%	512 (66)	424 (69)	88 (54)	
≥50%	269 (34)	194 (31)	75 (46)	
Creatinine (*n* = 699)	1.13 (0.96–1.41)	1.13 (0.97–1.41)	1.13 (0.93–1.39)	0.7
CRP (*n* = 723)	0.30 (0.14–0.88)	0.32 (0.15–0.84)	0.24 (0.11–0.95)	0.15
Haemoglobin (g/dl) (*n* = 777)	13.50 (12.00–14.70)	13.70 (12.10–14.97)	13.05 (11.20–14.10)	<0.001
Thrombocytes (/nl) (*n* = 726)	204 (167–249)	207 (169–250)	189 (161–240)	0.05
Leucocytes (/nl) (*n* = 776)	7.27 (6.04–8.78)	7.30 (6.12–8.79)	6.97 (5.88–8.65)	0.11
hs‐Troponin (pg/ml) (*n* = 441)	25 (16–47)	24 (16–46)	27 (17–48)	0.4
NT‐proBNP (pg/ml) (*n* = 559)	1367 (542–3289)	1307 (514–2940)	1726 (658–5172)	0.029
ACEi/ARB (*n* = 780)	609 (78)	481 (78)	128 (79)	0.9
ARNI (*n* = 781)	69 (9)	50 (8)	19 (12)	0.154
Aldosterone antagonist (*n* = 752)	275 (37)	222 (38)	53 (33)	0.3
Beta‐blocker (*n* = 750)	575 (77)	457 (77)	118 (74)	0.4
SGLT2i (*n* = 781)	66 (8)	54 (8)	12 (9)	0.12
Statin (*n* = 753)	617 (82)	482 (81)	135 (84)	0.4

Values are presented as *n* (%), or median (interquartile range).

ACEi, angiotensin‐converting enzyme inhibitor; ARNI, angiotensin receptor–neprilysin inhibitor; BMI, body mass index; COPD, chronic obstructive pulmonary disease; CRP, C‐reactive protein; hs‐high‐sensitivity; LOY, loss of Y chromosome; LVEF, left ventricular ejection fraction; NT‐proBNP, N‐terminal pro‐B‐type natriuretic peptide; SGLT2i, sodium–glucose co‐transporter 2 inhibitor; TAVR, transcatheter aortic valve replacement; VAF, variant allele frequency.

**Table 3:** Clinical characteristics of DNMT3A/TET2 clonal haematopoiesis of indeterminate potential (CHIP)‐driver mutation carriers with loss of Y chromosome (LOY) <17% compared to DNMT3A/TET2 CHIP‐driver mutation carriers with LOY ≥17%

Characteristic	DNMT3A/TET2 and LOY <17% (*n* = 127)	DNMT3A/TET2 and LOY ≥17% (*n* = 36)	*p*‐value
Cohort			
Ischaemic heart failure	43 (34)	6 (17)	0.047
TAVR	84 (66)	30 (83)	
DNMT3A VAF ≥2	77 (61)	16 (44)	0.083
TET2 VAF ≥2	58 (46)	22 (61)	0.1
COPD (*n* = 160)	16 (13)	3 (8.3)	0.6
Diabetes mellitus (*n* = 161)	44 (35)	14 (39)	0.7
Coronary artery disease (*n* = 161)	30 (83)	30 (83)	0.8
Hypertension (*n* = 161)	100 (80)	26 (72)	0.3
Atrial fibrillation (*n* = 160)	65 (52)	19 (53)	>0.9
History of smoking (*n* = 98)	43 (57)	15 (68)	0.3
Age, years	79 (72–83)	82 (76–86)	0.018
BMI, kg/m^2^ (*n* = 156)	26.7 (24.7–30.4)	26.7 (24.3–29.0)	0.5
LVEF, %	50 (32–60)	45 (34–58)	0.5
<50%	71 (56)	17 (47)	
≥50%	56 (44)	19 (53)	
Creatinine (*n* = 141)	1.13 (0.92–1.36)	1.15 (0.95–1.56)	0.7
CRP (*n* = 147)	0.28 (0.11–0.95)	0.16 (0.06–0.80)	0.2
Haemoglobin (g/dl) (*n* = 160)	13.10 (11.28–14.12)	12.90 (10.97–13.83)	0.5
Thrombocytes (/nl) (*n* = 153)	187 (160–244)	202 (164–230)	>0.9
Leucocytes (/nl) (*n* = 161)	6.88 (5.88–8.63)	7.12 (6.07–8.75)	0.9
hs‐Troponin (pg/ml) (*n* = 120)	28 (18–50)	24 (14–33)	0.11
NT‐proBNP (pg/ml) (*n* = 134)	1754 (654–5208)	1678 (806–4555)	>0.9
ACEi/ARB (*n* = 163)	96 (76)	32 (89)	0.086
ARNI (*n* = 163)	18 (14)	1 (2.8)	0.077
Aldosterone antagonist (*n* = 160)	44 (35)	9 (26)	0.4
Beta‐blocker (*n* = 159)	94 (75)	24 (71)	0.6
SGLT2i (*n* = 163)	22 (5.7)	6 (16.6)	0.927
Statin (*n* = 160)	105 (83)	30 (88)	0.5

Values are presented as *n* (%), or median (interquartile range).

ACEi, angiotensin‐converting enzyme inhibitor; ARNI, angiotensin receptor–neprilysin inhibitor; BMI, body mass index; COPD, chronic obstructive pulmonary disease; CRP, C‐reactive protein; hs, high‐sensitivity; LOY, loss of Y chromosome; LVEF, left ventricular ejection fraction; NT‐proBNP, N‐terminal pro‐B‐type natriuretic peptide; SGLT2i, sodium–glucose co‐transporter 2 inhibitor; TAVR, transcatheter aortic valve replacement; VAF, variant allele frequency.

### Prevalence of loss of Y chromosome and DNMT3A/TET2 CHIP‐driver mutations

**Figure 1:**
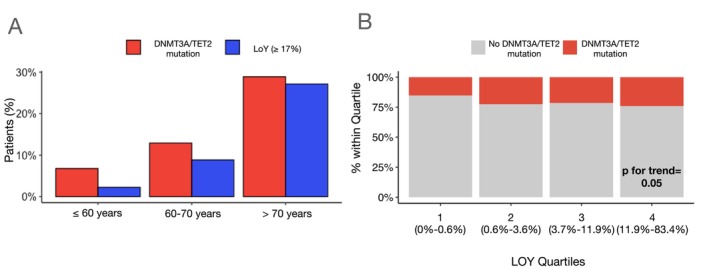
(*A*) Prevalence of loss of Y chromosome (LOY) ≥17% and DNMT3A/TET2 clonal haematopoiesis of indeterminate potential (CHIP)‐driver mutations according to age. (*B*) Prevalence of DNMT3A/TET2 CHIP‐driver mutations in LOY quartiles. The range of LOY within each quartile is indicated. Cochran–Armitage test for trend (*Z* = 1.95, *p* = 0.05).

As illustrated in *Figure* *1A*, both LOY and DNMT3A/TET2 CHIP‐driver mutations increased with age. Above the age of 70 years, 124 patients (27.1%) showed LOY ≥17%, whereas 132 patients (28.9%) carried DNMT3A and/or TET2 CHIP‐driver mutations. Importantly, the prevalence of harbouring DNMT3A/TET2 CHIP‐driver mutations significantly increased from 15.3% in patients in the lowest quartile of the extent of LOY up to 24.1% in the highest quartile of the extent of LOY (*Figure* *1B*). When characterizing the 141 patients with LOY ≥17%, 36 patients (26%) simultaneously harboured DNMT3A/TET2 CHIP‐driver mutations (*Table* *3*). There was no significant correlation between the extent of LOY and the VAF for both, DNMT3A and TET2 CHIP‐driver mutations (online supplementary *Figure Appendix* *S1*).

### Prognostic significance of loss of Y chromosome and DNMT3A/TET2 CHIP‐driver mutations

**Figure 2:**
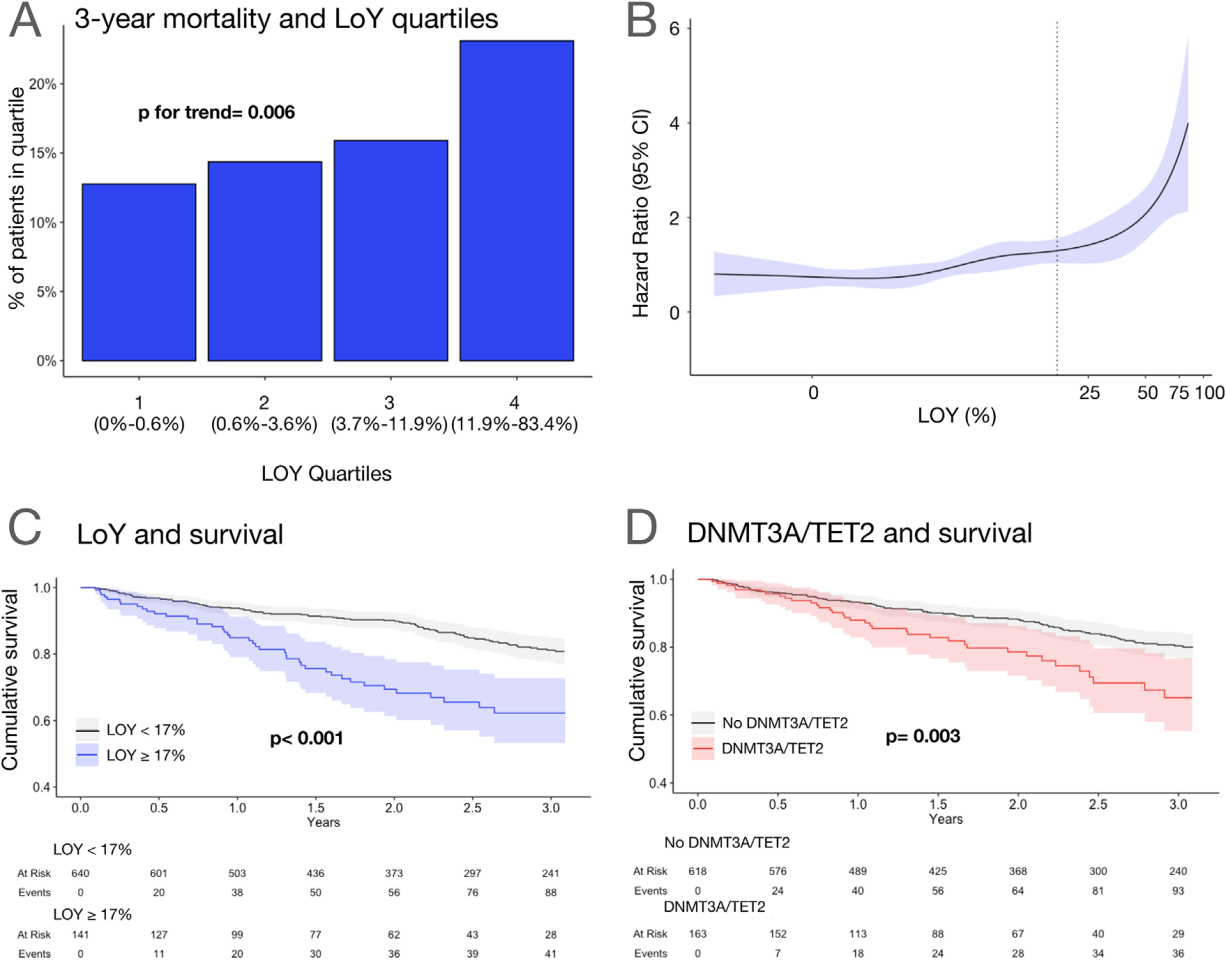
(*A*) Three‐year mortality according to loss of Y chromosome (LOY) quartiles. Increase in mortality was significant. The range of LOY within each quartile is indicated. Cochran–Armitage test for trend (*Z* = 2.73, *p* = 0.006). (*B*) Extent of LOY as continuous variable and hazard ratios for all‐cause mortality according to a cubic spline regression model, shaded areas represent 95% confidence intervals (CI), dashed line denotes LOY = 17%. (*C*) Kaplan–Meier survival curves in patients with LOY ≥17% compared to patients with LOY <17%. (*D*) Kaplan–Meier survival curves of patients harbouring DNMT3A/TET2 clonal haematopoiesis of indeterminate potential (CHIP)‐driver mutations compared to patients without DNMT3A/TET2 CHIP‐driver mutations.

The clinical characteristics and laboratory parameters of the 141 patients (18.1% of the whole cohort) with LOY ≥17% are compared with those of the patients with LOY <17% in *Table* *1*. As expected, patients with LOY ≥17% were significantly older, but also had significantly higher NT‐proBNP serum levels and higher values of LVEF as well as lower haemoglobin levels and a slightly reduced incidence of coronary artery disease, while the prevalence of DNMT3A/TET2 CHIP‐driver mutations was not significantly increased. All other measured parameters including kidney function, chronic obstructive pulmonary disease and diabetes did not differ. There was a dose‐dependent significant increase in mortality with increasing extent of LOY ranging from 12.8% in the lowest quartile to 23.1% in the highest quartile of LOY (*Figure* *2A*). Moreover, Cox regression analysis revealed that LOY as a continuous variable is a significant predictor of death in patients with heart failure (hazard ratio [HR] 1.02, 95% CI 1.01–1.03; *p* < 0.001) with a sharp increase in HR for death at LOY ≥17% (*Figure* *2B*).

During 3 years of follow‐up, 41 of 141 patients (29.1%) showing LOY ≥17% compared to 88 of 640 patients (13.8%) with LOY <17% died. *Figure* *2C* illustrates the Kaplan–Meier survival curves of the two cohorts demonstrating the significantly increased mortality in men having ≥17% LOY in circulating blood cells. Similarly, as previously reported,^2^, ^4^ harbouring a DNMT3A/TET2 CHIP‐driver mutation with a VAF ≥2% conferred an increased risk of death during follow‐up (*Figure* *2D*).

**Figure 3:**
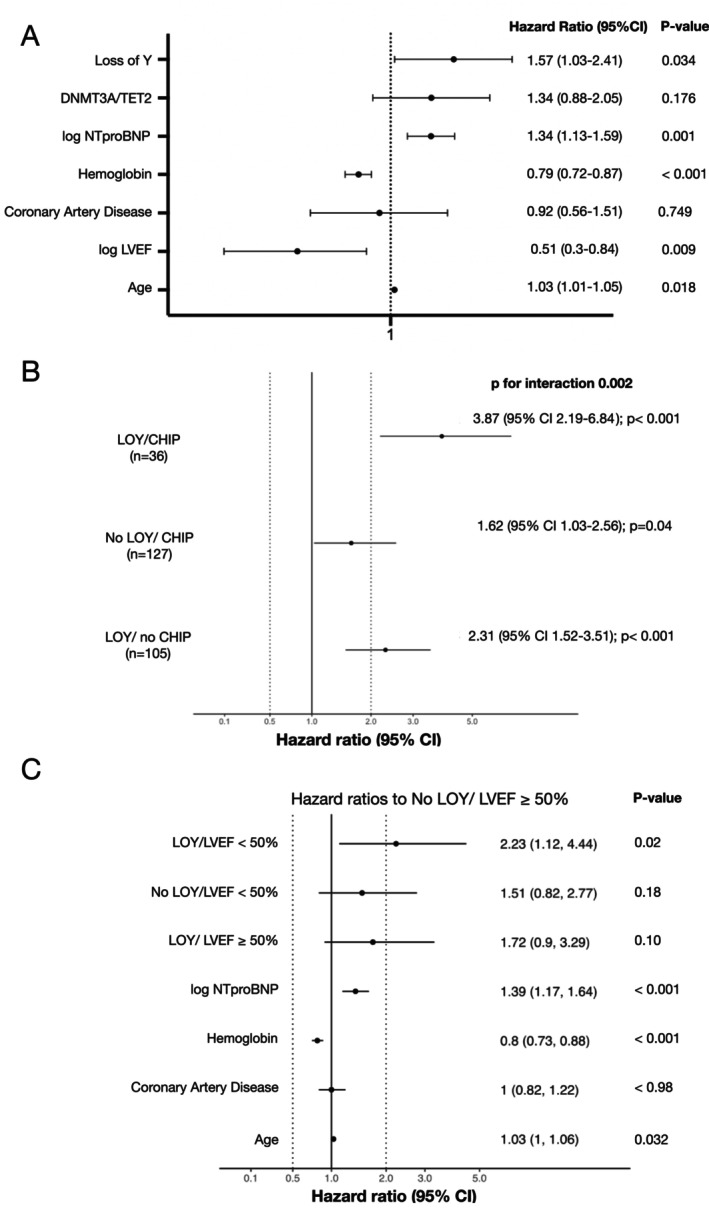
(*A*) Multivariate analysis of all‐cause mortality. Hazard ratio and 95% confidence intervals (CI) are displayed. (*B*) Univariate hazard ratios for all‐cause mortality for different groups according to the presence of either loss of Y chromosome (LOY) or DNMT3/TET2 clonal haematopoiesis of indeterminate potential (CHIP)‐driver mutations or both, LOY and DNMT3/TET2 CHIP‐driver mutations. Hazard ratio and 95% confidence intervals (CI) are displayed. Numbers for each group are indicated. A total of 513 men neither harboured LOY ≥17% nor DNMT3A/TET2 CHIP‐driver mutations. (*C*) Multivariate analysis of all‐cause mortality in a stratified Cox model with the sub‐ HFrEF and post‐TAVR cohorts being included as stratified variables. Hazard ratio and 95% CI compared to patients with LOY <17% and left ventricular ejection fraction (LVEF) ≥50% are displayed.

On multivariate analysis including all differentially distributed baseline characteristics (NT‐proBNP, age, LVEF, haemoglobin, and coronary artery disease), in addition to age, LVEF, NT‐proBNP and haemoglobin levels, LOY ≥17%, but not the presence of DNMT3A/TET2 CHIP‐driver mutations remained a significant independent predictor of mortality during follow‐up (*Figure* *3A*). Thus, LOY ≥17% appears to be independently associated with increased mortality during follow‐up irrespective of the presence of DNMT3A/TET2 CHIP‐driver mutations. As illustrated in *Figure* *3B*, harbouring only DNMT3A/TET2 CHIP‐driver mutations, but no LOY ≥17% was associated with a HR of 1.62 (95% CI 1.03–2.56), while harbouring LOY ≥17%, but no DNMT3A/TET2 CHIP‐driver mutation resulted in a HR of 2.31 (95% CI 1.52–3.51) and the presence of both, LOY ≥17% and DNMT3A/TET2 CHIP‐driver mutations, gave the highest HR for mortality with 3.87 (95% CI 2.19–6.84). However, since the patient cohort with preserved LVEF >50% was mainly composed of the significantly older post‐TAVR patients, we adjusted our further analyses by a stratified Cox model with the sub‐cohorts HFrEF and post‐TAVR being included as stratified variables (online supplementary *Figure* *S2*), which confirmed our results.

Statistical analysis demonstrated a significant interaction (*p* = 0.002) between the different HR indicating a significant effect of harbouring LOY ≥17% over and above DNMT3A/TET2 CHIP‐driver mutations on mortality (*Figure* *3B*). HRs did not differ with respect to smaller (VAF 2–10%) or larger CHIP‐mutation clone sizes (≥10%) (online supplementary *Figure* *S3A*). Multivariate analysis including NT‐proBNP, LVEF as a continuous variable, haemoglobin levels, presence of coronary artery disease and age revealed that LOY ≥17 is an independent predictor of mortality independent of ejection fraction (HR 1.52, 95% CI 1–2.31; *p* = 0.05) (online supplementary *Figure* *S3B*). Importantly, however, using the sub‐cohort adjusted stratified multivariable Cox model including NT‐proBNP, haemoglobin levels, presence of coronary artery disease and age revealed that LOY remained an independent predictor of mortality preferentially in patients with HFrEF, whereas statistical significance was narrowly missed in patients with an LVEF ≥50% (*Figure* *3C*). Baseline characteristics for patients with LVEF ≥50% and LVEF <50% are reported in online supplementary *Tables* *S3* and *S4*, respectively.

Importantly, except being slightly older, there were no important differences in clinical characteristics or laboratory parameters in DNMT3A/TET2 CHIP‐driver mutation carriers with or without LOY ≥17% (*Table* *3*).

Thus, LOY ≥17%, which co‐occurs with DNMT3A/TET2 CHIP‐driver mutations in more than 20% of all cases studied and occurs in 27.1% of all patients at the age above 70 years, appears to be an independent predictor of mortality in patients with HFrEF and is a major determinant of increased mortality in DNMT3A/TET2 CHIP‐driver mutation carriers with chronic heart failure.

### Validation of loss of Y chromosome to associate with mortality in patients with heart failure with reduced ejection fraction

**Figure 4:**
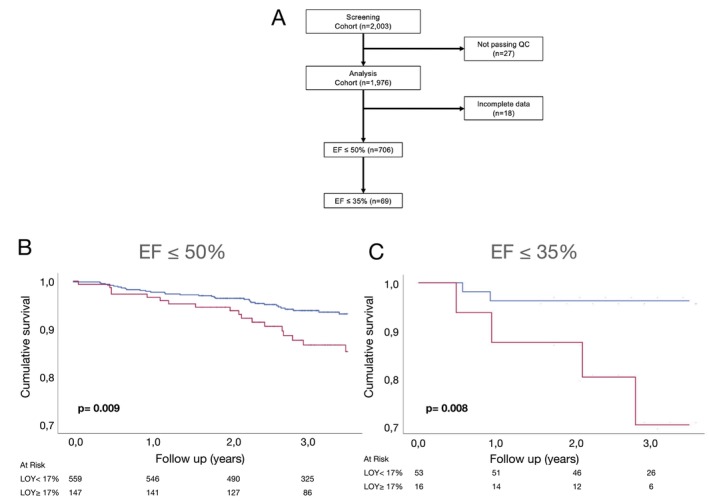
(*A*) Flow diagram showing selection of patient samples from the replication cohort for further analysis. (*B*) Kaplan–Meier survival curves in patients with left ventricular ejection fraction (EF) ≤50% and loss of Y chromosome (LOY) ≥17%, compared to LOY <17%. (*C*) Kaplan–Meier survival curves in patients with left ventricular EF ≤35% and LOY ≥17%, compared to LOY <17%. QC, quality control.

We finally validated the main findings of our study in an independent replication cohort of a total of 2003 male patients undergoing coronary angiography at the Heart Centre Munich. In this slightly younger cohort, 706 patients had a LVEF ≤50% and 147 (20.8%) of those patients had LOY ≥17% (*Figure* *4A*). There were no significant differences in age, laboratory parameters or comorbidities (*Table* *4*). As illustrated in *Figure* *4B*, Kaplan–Meier curve analysis confirmed the significantly reduced survival for patients with LOY ≥17%, which was most prominent in those patients with the lowest LVEF ≤35% (*Figure* *4C*). Baseline characteristics of patients with a LVEF ≤35% are displayed in *Table* *5*. Cox regression analysis revealed a HR for mortality of 2.12 (95% CI 1.19–3.77; *p* = 0.010) for patients with HFrEF and LOY.

**Table 4:** Baseline characteristics of all patients of the replication cohort with left ventricular ejection fraction ≤50% (*n* = 706) and patients with loss of Y chromosome <17% compared to ≥17%

Characteristic	Total (*n* = 706)	LOY <17% (*n* = 559)	LOY ≥17% (*n* = 147)	*p*‐value
DNMT3A VAF ≥2	80 (11.33)	63 (11.27)	17 (11.56)	0.885
TET2 VAF ≥2	57 (8.07)	42 (7.51)	15 (10.20)	0.307
DNMT3A and/or TET2	131 (18.56)	100 (17.89)	31 (21.09)	0.404
COPD (*n* = 674)	30 (4.45)	25 (4.70)	5 (3.52)	0.652
Diabetes mellitus (*n* = 674)	195 (28.93)	157 (29.51)	38 (26.76)	0.603
Hypertension (*n* = 674)	575 (85.31)	454 (85.34)	121 (85.21)	1000
Atrial fibrillation (*n* = 674)	48 (7.12)	41 (7.71)	7 (4.93)	0.357
History of smoking (*n* = 651)	285 (42.28)	218 (42.17)	67 (50.00)	0.118
Hyperlipidaemia	616 (87.25)	484 (86.58)	132 (89.80)	0.333
Age, years	71 (61–79)	72 (62–79)	70 (61–78)	0.368
BMI, kg/m^2^ (*n* = 665)	27.40 (24.86–30.42)	27.47 (24.86–30.42)	26.99 (24.90–30.86)	0.572
LV function (*n* = 706)				
≥35%–≤50%	637 (90.23)	506 (90.52)	131 (89.12)	0.640
<35%	69 (9.77)	53 (9.48)	16 (10.88)	
Creatinine (*n* = 700)	1.04 (0.91–1.21)	1.06 (0.93–1.22)	1.01 (0.87–1.16)	0.070
CRP (*n* = 700)	1.47 (0.72–3.46)	1.43 (0.72–3.43)	1.35 (0.69–2.83)	0.243
Haemoglobin (g/dl) (*n* = 700)	14.2 (13.1–15.1)	14.10 (13.10–15.00)	14.35 (13.30–15.40)	0.142
Thrombocytes (/nl) (*n* = 700)	208 (174–250)	206 (173–247.50)	216 (182–258)	0.144
Leucocytes (/nl) (*n* = 700)	7.1 (6.09–8.64)	7.06 (6.06–8.59)	7.20 (6.27–8.70)	0.325
hs‐Troponin (pg/ml) (*n* = 700)	22.5 (13–47)	24 (14–48)	21 (11.5–37)	0.050
NT‐proBNP (pg/ml) (*n* = 250)	973 (336–2335)	1175 (338–2570)	618 (321–1180)	0.143

Values are presented as *n* (%), or median (interquartile range).

BMI, body mass index; COPD, chronic obstructive pulmonary disease; CRP, C‐reactive protein; hs, high‐sensitivity; LOY, loss of Y chromosome; LV, left ventricular; NT‐proBNP, N‐terminal pro‐B‐type natriuretic peptide; VAF, variant allele frequency.

**Table 5:** Baseline characteristics of all patients of the replication cohort with left ventricular ejection fraction ≤35% (*n* = 69) and patients with loss of Y chromosome <17% compared to ≥17%

Characteristic	Total (*n* = 69)	LOY <17% (*n* = 53)	LOY ≥17% (*n* = 16)	*p*‐value
DNMT3A	3 (4.35)	2 (3.77)	1 (6.25)	0.553
TET2	9 (13.04)	5 (9.43)	4 (25.00)	0.197
DNMT3A or TET2	11 (15.94)	6 (11.32)	5 (31.25)	0.111
COPD (*n* = 66)	1 (1.52)	0 (0.00)	1 (6.25)	0.212
Diabetes mellitus (*n* = 66)	17 (25.76)	15 (28.30)	2 (12.50)	0.327
Hypertension (*n* = 66)	58 (87.88)	44 (83.02)	14 (87.50)	0.187
Atrial fibrillation (*n* = 66)	5 (7.58)	5 (9.43)	0 (0.00)	0.576
History of smoking (*n* = 63)	23 (36.51)	18 (33.96)	5 (31.25)	1000
Hyperlipidaemia	60 (86.96)	46 (86.79)	14 (87.50)	1000
Age, years	70 (62–77)	68 (62–78)	70 (66–75)	0.422
BMI, kg/m^2^ (*n* = 65)	27.41 (24.91–31.22)	27.37 (24.48–31.18)	28.10 (26.25–31.35)	0.539
Creatinine (*n* = 69)	1.00 (0.87–1.20)	1.02 (0.86–1.15)	0.98 (0.82–1.25)	0.386
CRP (*n* = 69)	1.57 (0.91–3.35)	1.60 (0.94–3.31)	1.23 (0.60–1.84)	0.526
Haemoglobin (g/dl) (*n* = 69)	14.30 (13.50–15.10)	14.30 (13.50–14.90)	14.40 (13.50–15.80)	0.754
Thrombocytes (/nl) (*n* = 69)	223 (193–270)	219 (193–268)	240 (193–278)	0.599
Leucocytes (/nl) (*n* = 69)	6.85 (6.06–8.88)	6.83 (5.98–8.81)	6.70 (5.79–8.70)	0.926
hs‐Troponin (pg/ml) (*n* = 69)	17.00 (12.00–32.00)	21 (12–37)	17.00 (10.50–24.50)	0.438
NT‐proBNP (pg/ml) (*n* = 23)	505 (336–1545)	495 (249–988)	6080 (3209–6585)	0.309

Values are presented as *n* (%), or median (interquartile range).

BMI, body mass index; COPD, chronic obstructive pulmonary disease; CRP, C‐reactive protein; hs, high‐sensitivity; LOY, loss of Y chromosome; NT‐proBNP, N‐terminal pro‐B‐type natriuretic peptide.

Thus, the results of our replication cohort confirm that LOY is an independent predictor of death in patients with HFrEF.

### Potential mechanistic insights by single‐cell RNA sequencing analyses of blood cells

**Figure 5:**
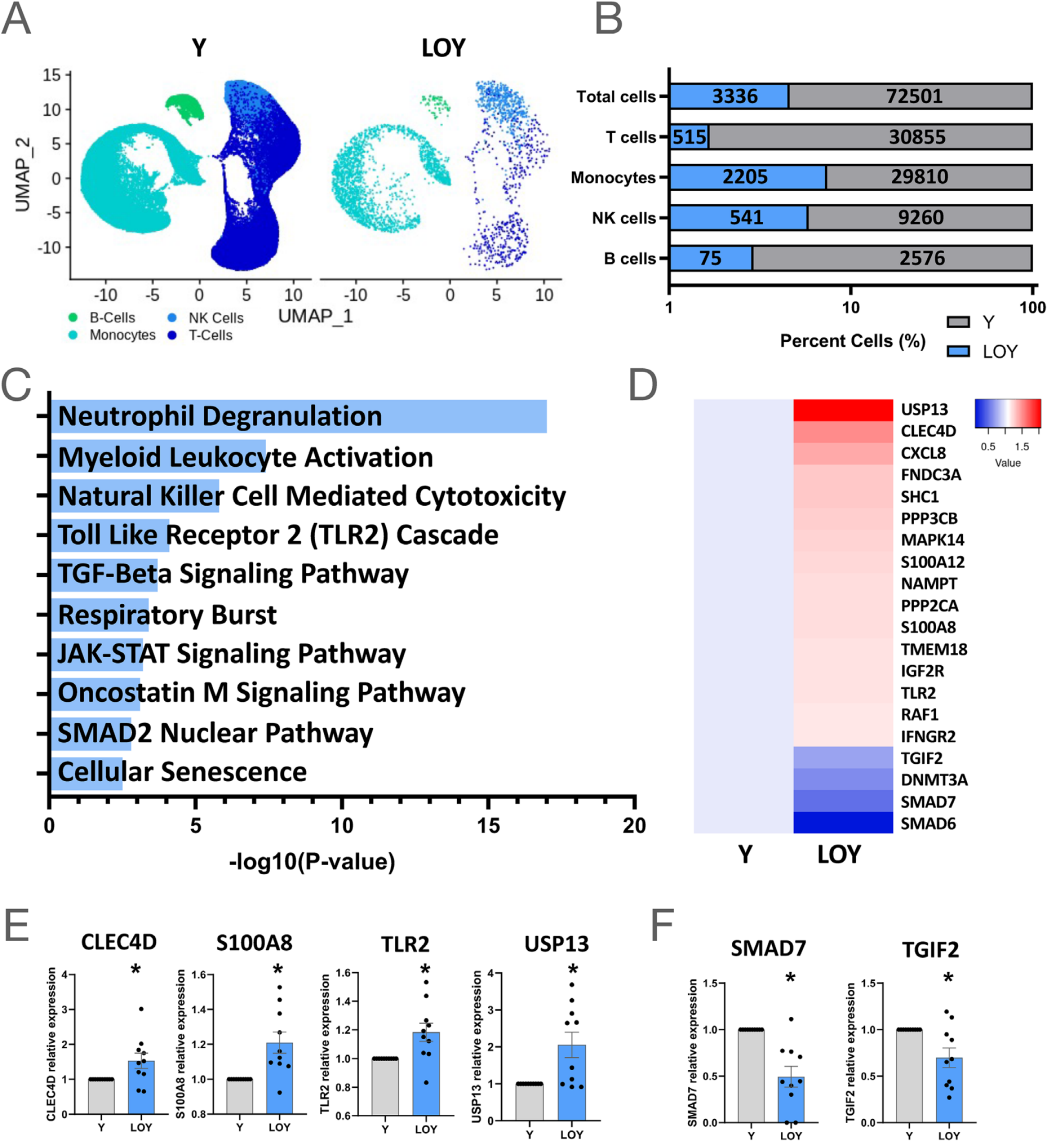
Single‐cell RNA sequencing analysis of loss of Y chromosome (LOY) cell distribution and monocytic profiles in heart failure patients shows pro‐fibrotic signature. (*A*) Uniform manifold approximation and projection (UMAP) of peripheral blood mononuclear cells. (*B*) Percent LOY and Y‐harbouring cells by cell type with absolute cell counts (*n* = 10). (*C*) Gene ontology terms associated with LOY‐regulated genes. (*D*) Heatmap of regulated genes in LOY monocytes associated with monocyte activation, fibrosis and transforming growth factor‐β signalling. (*E*, *F*) Relative expression of (*E*) up‐regulated and (*F*) down‐regulated genes by bar plot. Panels *E*, *F*: *n* = 10; significance: *p* < 0.05 by paired *t*‐test.

To delineate transcriptional differences in circulating haematopoietic cells with and without LOY and to assess the effect of the presence of CHIP mutations on LOY cells, we analysed recently published scRNA‐seq data derived from circulating peripheral blood mononuclear cells of 10 HFrEF patients of the present cohort (mean age of 67.3 years and ejection fraction of 34.4%).^20^ LOY was defined in scRNA‐seq data as combinatorial loss of expression of any gene encoded on the Y chromosome. There was a close correlation between droplet digital PCR determined results for the extent of LOY and percentage of Y‐deficient cells in scRNA‐seq analysis (slope 1.04, Pearson *r* = 0.8, *p* = 0.0019). As illustrated in *Figure* *5A*, LOY cells were intermingled with Y chromosome‐encoded genes expressing cells and did not form a specific cluster of cells. Assessing the relative distribution of LOY in different peripheral blood cells revealed that monocytes exhibited the greatest absolute count of LOY cells (*Figure* *5B*). Given the abundance of monocytic LOY cells and the previously reported impact on cardiac fibrosis, we focused on monocytes for further analysis. Gene ontology (GO) terms derived from up‐regulated genes in LOY monocytes were associated with macrophage‐mediated tissue damage and cardiac fibrosis with terms such as ‘Toll‐like receptor 2 (TLR2) cascade’ and ‘transforming growth factor (TGF)‐β signalling pathway’ (*Figure* *5C*). Focusing on significantly regulated genes taken from GO terms, the expression heatmap shown in *Figure* *5D* demonstrates a pronounced pro‐fibrotic, activated gene signature in LOY monocytes.

Out of the 193 up‐regulated genes in LOY monocytes, *Figure* *5E* illustrates examples of relative expression levels for prototypical pro‐fibrotic and tissue damage‐associated pathways such as TLR2, which promotes cardiac fibrosis via IRAK/NFkB signalling,^23^ and *S100A8, CLEC4D* and *USP13*, which promote fibrosis and tissue disruption as members and stabilizers of the damage‐associated molecular pattern family. Of the 1488 down‐regulated genes in LOY monocytes, the profoundly decreased expression levels of SMAD7 and TGIF2 are of special interest, as both restrain TGF‐β induced activation pathways (*Figure* *5F*). These data strongly suggest an increased capacity for pro‐fibrotic signalling in circulating monocytes lacking Y chromosome‐encoded genes.

**Figure 6:**
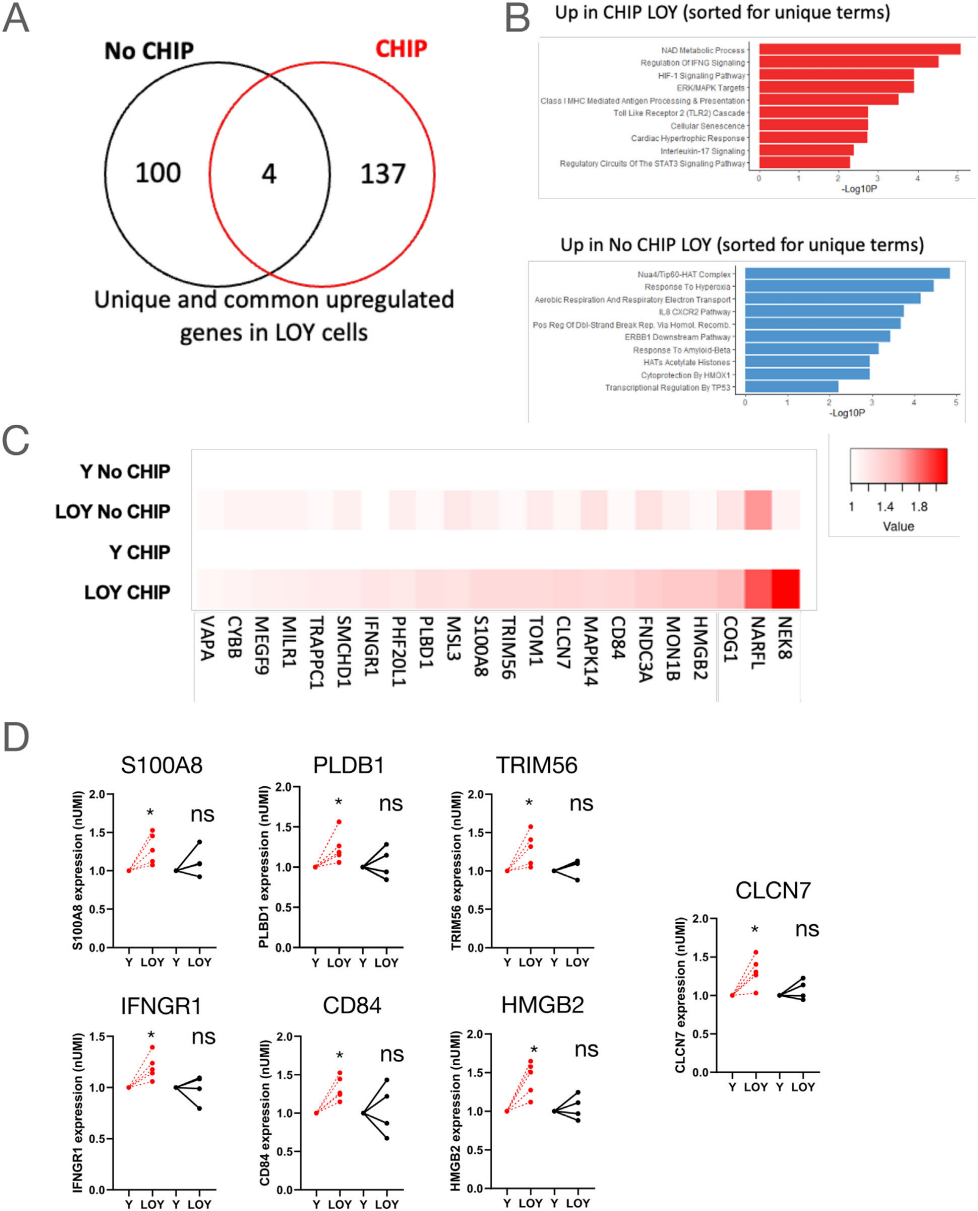
Patient‐level analysis comparing gene expression in patients with DNMT3A clonal haematopoiesis of indeterminate potential (CHIP) (*n* = 5) and no CHIP (*n* = 4). (*A*) Venn diagram displaying common and unique unregulated genes in loss of Y chromosome (LOY) cells compared to Y cells in patients with CHIP and no CHIP. (*B*) Gene ontology terms associated with LOY‐regulated genes in patients with CHIP and no CHIP. (*C*) Heatmap of regulated genes in LOY monocytes of patients with CHIP and no CHIP compared to Y cells. (*D*) Patient‐level analysis of regulated genes in LOY cells compared to Y cells in patients with CHIP and no CHIP. *p < 0.05 by paired *t*‐test.

In order to gain potential insights into the detrimental effect of the combination of both, the presence of harbouring CHIP mutations and LOY ≥17%, on prognosis in patients with heart failure, a paired analysis of LOY and Y cells in patients with and without DNMT3A mutations was performed. Among the 10 patients with HFrEF and available scRNA‐seq data, four patients did not have CHIP mutations, whereas five patients had DNMT3A CHIP‐driver mutations with a VAF ≥2%. One patient had a DNMT3A mutation with a VAF of 1.1% and was excluded from further analysis. Up‐regulated genes in LOY cells were mutually exclusive in patients with and without DNMT3A CHIP‐driver mutations (*Figure* *6A,B*). Specifically up‐regulated GO terms in LOY cells derived from patients simultaneously harbouring DNMT3A CHIP‐driver mutations were related to maladaptive inflammation such as ‘regulation of IFNG signalling’, ‘class I MHC‐mediated antigen processing and presentation’ as well as ‘TLR2 cascade’ and ‘cellular senescence’. In addition, pathways related to fibrosis such as ‘hypoxia‐inducible factor (HIF)‐1 signalling pathway’ and ‘ERK/MAPK targets’ or ‘cardiac hypertrophic response’ were regulated. In contrast, we did not find GO terms related to increased inflammation in LOY cells of patients without CHIP mutations compared to Y cells of the same patients (*Figure* *6C*). As shown in the heatmap in *Figure* *6D* and in a paired analysis in individual patients with and without DNMT3A mutations, pro‐inflammatory genes such as the *alarmins* S100A8, which initiates neutrophil chemotaxis as a damage‐associated molecular pattern, and HMGB2, which acts as pro‐inflammatory cytokine in the extracellular space and binds to RAGE to induce oxidative stress and inflammation, were significantly upregulated in LOY cells of patients simultaneously harbouring DNMT3A CHIP‐driver mutations. Moreover, IFNGR and CD84, which enhances interferon‐γ secretion,^24^ as well as the interferon‐inducible E3 ubiquitin ligase TRIM56, which modulates cGAS‐STING, a critical driver of ageing‐related inflammation, were specifically up‐regulated in LOY cells of patients with DNMT3A mutations. Finally, PLBD1, which generates lipid mediators of inflammation,^25^ and CLCN7, a major activator of cellular lysosomal activity,^26^, ^27^ were significantly up‐regulated only in LOY cells of patients with DNMT3A mutations (*Figure* *6E*). These data support the conclusion that, in addition to pro‐fibrotic activation, LOY monocytes of patients simultaneously harbouring DNMT3A CHIP‐driver mutations display genetic expression features of augmented generic paracrine and cell‐intrinsic inflammation pathways.

## Discussion

The present study is the first to investigate the prognostic significance of LOY, the most common acquired somatic mutation in human blood cells, in male patients with established chronic heart failure. Our results provide the following insights: (i) LOY is a major independent determinant of increased mortality specifically in patients with HFrEF; (ii) the age‐related prevalence of LOY parallels the age‐associated prevalence of DNMT3A/TET2 CHIP‐driver mutations in chronic heart failure; (iii) in men with chronic heart failure, a substantial number of patients harbouring CHIP‐driver mutations also show LOY ≥17%; (iv) the co‐occurrence of LOY in part contributes to the well‐established increased mortality observed in patients with chronic heart failure carrying DNMT3A/TET2 CHIP‐driver mutations; and (v) mechanistically, scRNA‐seq analyses of circulating monocytes lacking Y chromosome‐encoded genes exhibit profibrotic genetic signatures, which are further enhanced by features of augmented generic inflammation pathways in HFrEF patients simultaneously harbouring DNMT3A CHIP‐driver mutations. Thus, when addressing the prognostic significance of age‐related acquired somatic DNA mutations in circulating blood cells of men with chronic heart failure, it appears to be of utmost importance to account for the presence of LOY.

Loss of Y chromosome has been previously linked to shorter life span in elderly men and associated with age‐associated degenerative diseases like Alzheimer's disease, macular degeneration, and cardiovascular disease in large epidemiological studies.^12^, ^28^, ^29^, ^30^, ^31^ It was only until very recently that it was experimentally discovered in a mouse model mimicking LOY in haematopoietic stem cells that LOY led to diffuse cardiac fibrosis during ageing, which was accompanied by a progressive decline in cardiac function.^15^ This study indicated for the first time that LOY in haematopoietic cells was linked to heart failure.^32^ Mechanistically, modelling LOY in bone marrow cells led to polarization of bone marrow‐derived macrophages lacking the Y chromosome towards a pro‐fibrotic phenotype leading to fibroblast activation, deposition of excessive extracellular matrix and cardiac fibrosis in large part mediated via TGF‐β signaling pathways.^15^ We could recently extend these findings into the clinical scenario by studying a very elderly patient population of men undergoing TAVR for severe degenerative aortic valve stenosis.^16^ Using scRNA‐seq analysis of patient‐derived circulating blood cells, we could demonstrate that LOY in monocytes is associated with a pro‐fibrotic gene signature characterized by sensitizing the cells for the TGF‐β signalling pathway.^16^ As diffuse cardiac fibrosis represents the common denominator of progression of all forms of chronic heart failure,^33^ we hypothesized that the presence of LOY might associate with worse survival in patients with established chronic heart failure. Indeed, the results of the present study now disclose that LOY associates with impaired survival of men with chronic heart failure. Thus, our findings not only further affirm the clinical relevance of the experimental observations by Sano *et al*.,^15^ but may also open new perspectives on risk factors for worse outcomes as well as precision treatment strategies in men with chronic heart failure. Indeed, the results of the present study further establish a role for pro‐fibrotic signalling in LOY monocytes in patients with chronic heart failure suggesting that patients with substantial LOY may derive significant benefit from antifibrotic therapies.

As clonal haematopoiesis, another acquired somatic mosaic condition in circulating myeloid cells, shares many similarities with LOY, including its association with advanced age as well as its prognostic significance in patients with HFrEF,^2^, ^3^, ^4^, ^5^ we also investigated a potential interaction between LOY and CHIP in our patient cohort. Mechanistically, DNMT3A/TET2 CHIP‐driver mutations may exert their detrimental effects on cardiovascular disease predominantly by a pro‐inflammatory activation of circulating blood cells.^34^, ^35^, ^36^, ^37^ Specifically, patients with chronic heart failure display a pro‐inflammatory gene signature in their circulating myeloid cells, which is further aggravated in carriers of DNMT3A/TET2 CHIP‐driver mutations.^38^ In concordance with previous small studies, the prevalence of DNMT3A/TET2 CHIP‐driver mutations increased significantly with increasing extent of LOY.^17^, ^39^ Likewise, in carriers of DNMT3A/TET2 CHIP‐driver mutations, 24.3% of patients were simultaneously showing LOY ≥17%. Thus, there is considerable coexistence of LOY and CHIP in men with chronic heart failure, which is presumably due to the advanced age of the patients studied, as statistical testing failed to establish a significant association between the presence or extent of LOY and DNMT3A/TET2 CHIP‐driver mutations in our cohort.

Most importantly, the co‐occurrence of LOY ≥17% in patients harbouring DNMT3A/TET2 CHIP‐driver mutations in part contributed to the observed prognostic significance of carrying DNMT3A/TET2 CHIP‐driver mutations, while LOY ≥17% was associated with increased mortality during follow‐up irrespective of the presence of DNMT3A/TET2 CHIP‐driver mutations in patients with HFrEF. Notably, patients harbouring both, LOY ≥17% as well as DNMT3A/TET2 CHIP‐driver mutations, demonstrated the highest HR for mortality. Mechanistically, this observation may be well rationalized by the combination of pro‐inflammatory and pro‐fibrotic activation of circulating blood cells contributing to worse clinical outcome in patients with heart failure.^16^, ^34^, ^35^, ^36^, ^40^, ^41^, ^42^ Indeed, the results of our scRNA‐seq analysis extended our previously published observation in elderly patients undergoing TAVR^16^ by demonstrating that LOY in circulating monocytes of patients with chronic heart failure associates with an increased pro‐fibrotic gene signature. Specifically, expression levels of TGIF2 and SMAD7, which restrain TGF‐β induced activation pathways and prevent TGF‐β mediated fibrosis in post‐infarction failing hearts,^43^ were significantly down‐regulated, while expression of TLR2 and S100A8, which promote inflammation, fibrosis and progression of heart failure,^23^, ^44^, ^45^ were significantly induced in LOY monocytes.

More importantly, LOY monocytes in HFrEF patients simultaneously harbouring DNMT3A CHIP‐driver mutations demonstrated a boosted, more generic inflammatory activation genetic signature compared to LOY monocytes of patients harbouring no CHIP‐driver mutations. Here, expression of HMGB2 and PLBD1, which act pro‐inflammatory in a paracrine fashion either by acting as cytokine itself of by generating lipid mediators of inflammation, respectively, and were both shown to correlate with cardiac functional deterioration post‐infarction,^25^, ^46^, ^47^ were specifically up‐regulated in LOY monocytes of patients simultaneously harbouring DNMT3A CHIP‐driver mutations. Moreover, expression of TRIM56 and IFNGR1, which both act as mediators of immune dysregulation by activating the cGAS‐STING signalling pathway,^48^, ^49^ a critical driver of ageing‐related inflammation^50^ and known to contribute to adverse left ventricular remodelling leading to heart failure in mice,^51^ were specifically up‐regulated in LOY monocytes of patients simultaneously harbouring DNMT3A CHIP‐driver mutations.

In conclusion, the complex relationship between different classes of age‐related somatically acquired mutations needs to be considered when assessing clinical outcomes. At least for male patients with chronic heart failure, LOY ≥17% appears to be an independent risk factor for increased mortality, and its co‐occurrence with CHIP may partly confound the prognostic significance of DNMT3A/TET2 CHIP‐driver mutations.

### Limitations

The present clinical study cannot provide a cause‐and‐effect relationship between LOY and increased mortality in patients with chronic heart failure. As such, we can only conclude that LOY is an important independent predictor of mortality in HFrEF. In addition, we can only report all‐cause mortality data, but cannot provide data on cause‐specific mortality or hospitalizations for heart failure. However, we have recently shown that LOY ≥17% associates with incident heart failure events including progression of systolic and diastolic dysfunction, hospitalization for heart failure and cardiovascular death in patients with chronic kidney disease.^52^ In addition, the results of our replication cohort demonstrated that all‐cause mortality was most accentuated in patients with the most severely depressed LVEF suggesting that our observations on all‐cause mortality do not merely reflect the well‐known association of LOY with shorter life span but may indeed be specific for the setting of chronic heart failure. Moreover, our findings may only relate to patients with established chronic heart failure but may not be applicable to other forms of cardiovascular disease or incident heart failure. In addition, patients with HFpEF of the present study do not represent the classical HFpEF population, but rather represent a rather specific very elderly cohort of patients with persistent symptoms and signs of heart failure secondary to aortic stenosis‐induced left ventricular hypertrophy even after successful removal of the stenotic valve by TAVR. Indeed, while sub‐analysis excluding patients post‐TAVR confirmed the prognostic significance of LOY, it became apparent that LOY appears to be specifically relevant as an independent predictor of mortality in patients with HFrEF, which was also confirmed in our replication cohort. Thus, further studies are required to document an independent prognostic role of LOY in patients with typical HFpEF. We used an Youden index‐derived optimal cut‐off of 17% to assess the effects of LOY on mortality. As in our previous report in elderly patients with severe aortic valve stenosis undergoing TAVR,^16^ there was a dose‐dependent increase in mortality with increasing extent of LOY with a sharp increase in the HRs for death at LOY ≥17%. In contrast, for DNMT3A/TET2 mutations, we did not select an optimized cut‐off level, but rather used the generally accepted definition of CHIP as ≥2% VAF. However, using larger clone sizes of CHIP with VAF >10% did not alter the results. Our scRNA‐seq analyses of circulating monocytes only refer to LOY in HFrEF patients with or without simultaneously harbouring DNMT3A CHIP‐driver mutations. Thus, although it has recently been reported that the enhanced inflammation in loss of Dnmt3a in myeloid cells experimentally phenocopies the effect of loss of Tet2 in a mouse model of atherosclerosis,^41^ we cannot comment on potential combinatorial effects of LOY and TET2 CHIP‐driver mutations in patients with chronic heart failure. We primarily focused on the two most common CHIP‐driver mutations, DNMT3A and TET2, for which a prognostic role in heart failure is firmly established. Thus, we cannot comment on other pathogenic CHIP‐driver mutations implicated in cardiovascular diseases. Finally, mortality rates were significantly lower in our validation cohort, which may have affected the statistical significance of the analysis. However, HRs for the prognostic relevance of harbouring LOY were comparable in the initial cohort and in the replication cohort.

## Supporting information


**Appendix S1.** Supporting information.
